# Direct Coronary Artery Reimplantation of Anomalous Aortic Origin of the Right Coronary Artery: A Case Report

**DOI:** 10.7759/cureus.45205

**Published:** 2023-09-14

**Authors:** Yusuke Katoh, Satoshi Ohki, Ryo Yamaguchi, Takao Miki, Ayako Nagasawa, Shuichi Okonogi, Kiyomitsu Yasuhara, Tamiyuki Obayashi

**Affiliations:** 1 Cardiovascular Surgery, Isesaki Municipal Hospital, Isesaki, JPN

**Keywords:** coronary artery anomaly, anomalous aortic origin of the coronary artery, right coronary artery reimplantation, congenital heart disease, anomalous aortic origin of the right coronary artery

## Abstract

Anomalous aortic origin of the coronary artery (AAOCA) is a rare congenital cardiac abnormality. Although AAOCA can cause angina, syncope, palpitations, and sudden cardiac death, most patients remain asymptomatic. A 60-year-old woman experienced occasional chest discomfort. A coronary computed tomography (CT) showed that the right coronary artery (RCA) originated from the left sinus of Valsalva, indicating AAORCA. Exercise myocardial scintigraphy revealed ischemia in the inferior wall. Cardiac catheterization showed stenosis in the ostium of the RCA. Therefore, direct reimplantation of the RCA into the right sinus was performed under cardiopulmonary bypass. The patient recovered uneventfully, postoperatively. Postoperative coronary CT showed no evidence of bending or stenosis in the RCA. Moreover, exercise scintigraphy showed no ischemic changes. The patient was discharged on postoperative day 18 after the resolution of chest discomfort and remained healthy for the following one year. AAORCA is a rare congenital abnormality that could lead to sudden cardiac death. Appropriate imaging studies and surgery should be performed in symptomatic patients with AAORCA who have inter-arterial paths between the ascending aorta and pulmonary artery with right coronary ostial stenosis. Reimplantation of the RCA directly into the right coronary sinus with adequate mobilization of the RCA is a simple procedure that can return the anatomic and biophysiologic status of AAORCA patients to normal and resolve most morphologic abnormalities.

## Introduction

Anomalous aortic origin of the coronary artery (AAOCA) is a rare congenital cardiac abnormality that typically involves the left main coronary artery arising from the right sinus of Valsalva or the right coronary artery (RCA) arising from the left sinus [1,2]. In our case, coronary computed tomography (CT) showed that the RCA originated from the left sinus of Valsalva, indicating AAORCA. The proximal course of the RCA ran between the ascending aorta and pulmonary artery (inter-arterial) to reach the myocardium. Preoperative RCA angiography revealed stenosis in the ostium. A symptomatic or proven myocardial ischemic patient is indicated for surgery. Therefore, we performed direct reimplantation of the RCA into the right sinus of Valsalva [3]. The location of the neo-ostium of the RCA was determined using transesophageal echocardiography (TEE) during surgery.

## Case presentation

**Figure 1:**
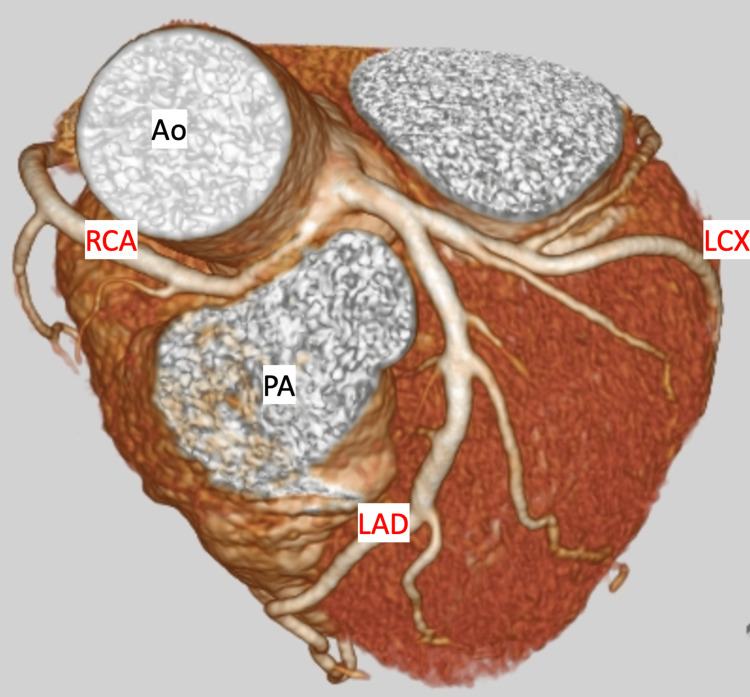
Preoperative coronary computed tomography The right coronary artery originated from the left sinus of Valsalva and it traveled between the ascending aorta and pulmonary artery. LAD, left anterior descending artery; LCX, left circumflex artery; RCA, right coronary artery; Ao, aorta; PA, pulmonary artery.

A 60-year-old woman presented with a history of chest discomfort. Electrocardiography, Holter electrocardiography, and blood test results revealed no abnormal findings. A coronary CT showed that the RCA originated from the left sinus of Valsalva, indicating AAORCA. The RCA traveled between the ascending aorta and pulmonary artery (Figure 1).

**Figure 2:**
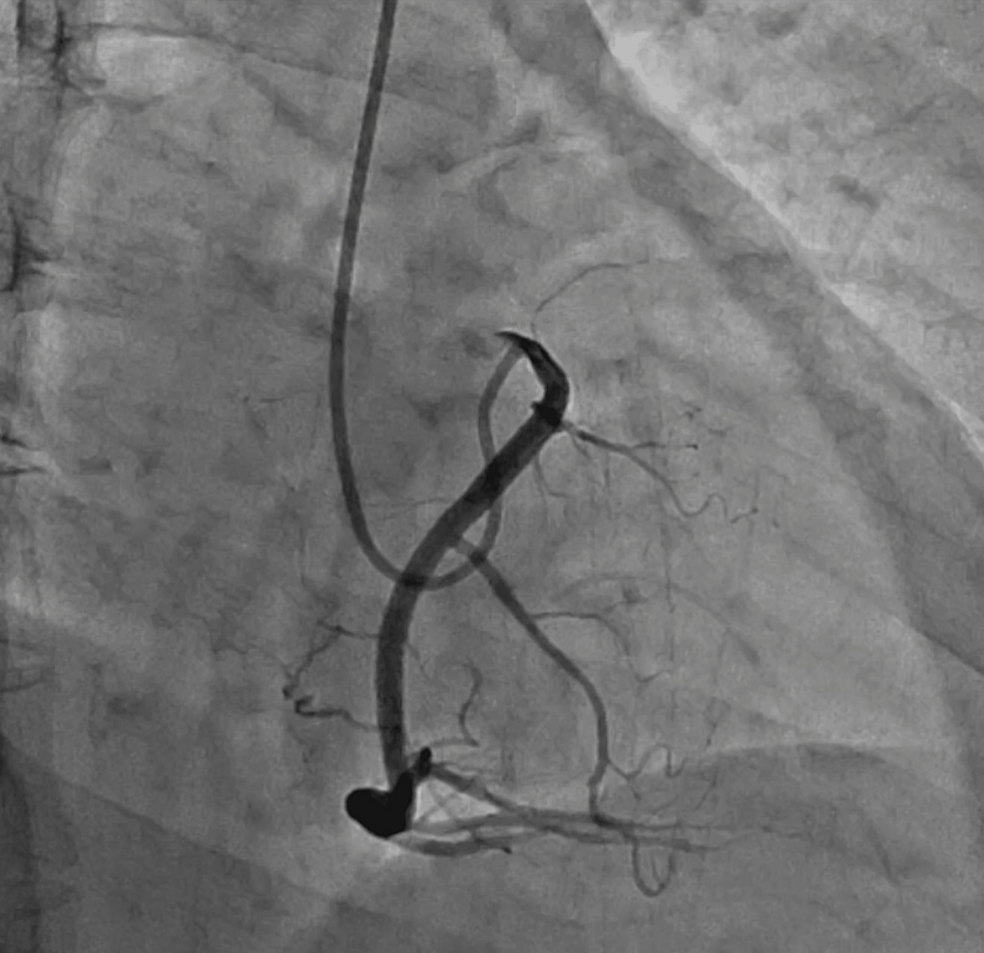
Preoperative right coronary artery angiography It showed stenosis in the ostium.

Although cardiac catheterization revealed an intact left coronary artery, no coronary artery originating from the right sinus of Valsalva was found. Instead, the RCA originated from the left sinus with stenosis in the ostium (Figure 2).

**Figure 3:**
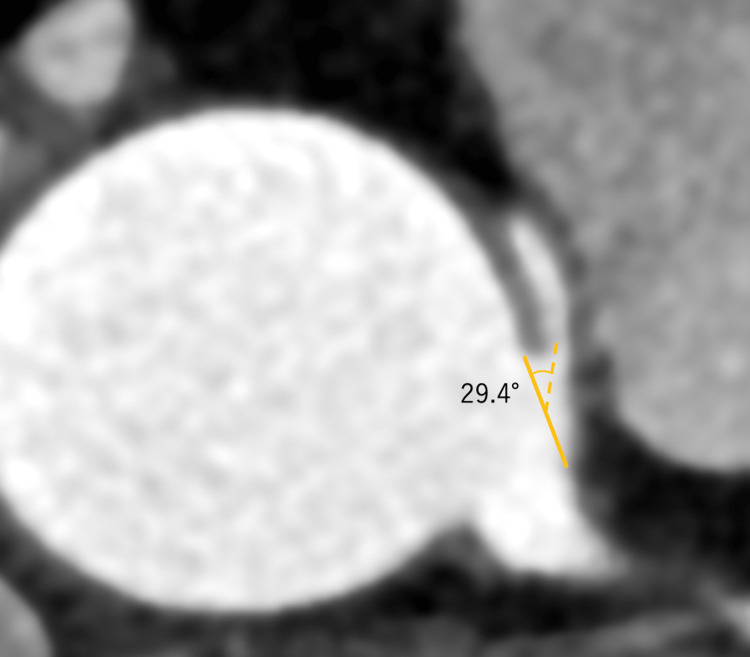
Contrast CT horizontal section at the level of the right coronary bifurcation The solid line is the ostium line, and the dashed line is the line drawn from the midpoint of the ostium line to a point 5 mm along the center of the coronary artery origin. The angle of separation is 29.4°.

The right and left coronary arteries were in close proximity to each other but had separate arterial outlets. The steep angle at the coronary artery inlet was defined as an angle of less than 45° between the plane formed by a point 5 mm along the vessel centerline from the ostium center and the plane tangential to the aorta in CT slices at the RCA ostium level [4]. The take-off angle of the AAORCA from the ascending aorta was 29.4° in our case (Figure 3).

**Figure 4:**
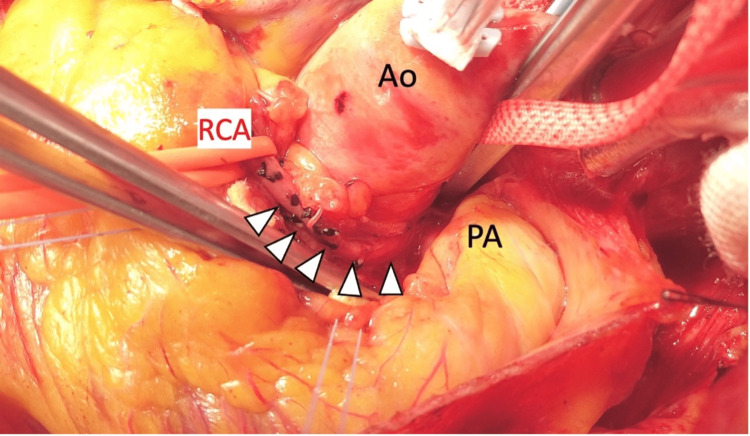
Intraoperative photograph The ascending aorta and pulmonary artery are dissected, and the right coronary artery is dissected close to the root. Ao, aorta; PA, pulmonary artery; RCA, right coronary artery.

**Figure 5:**
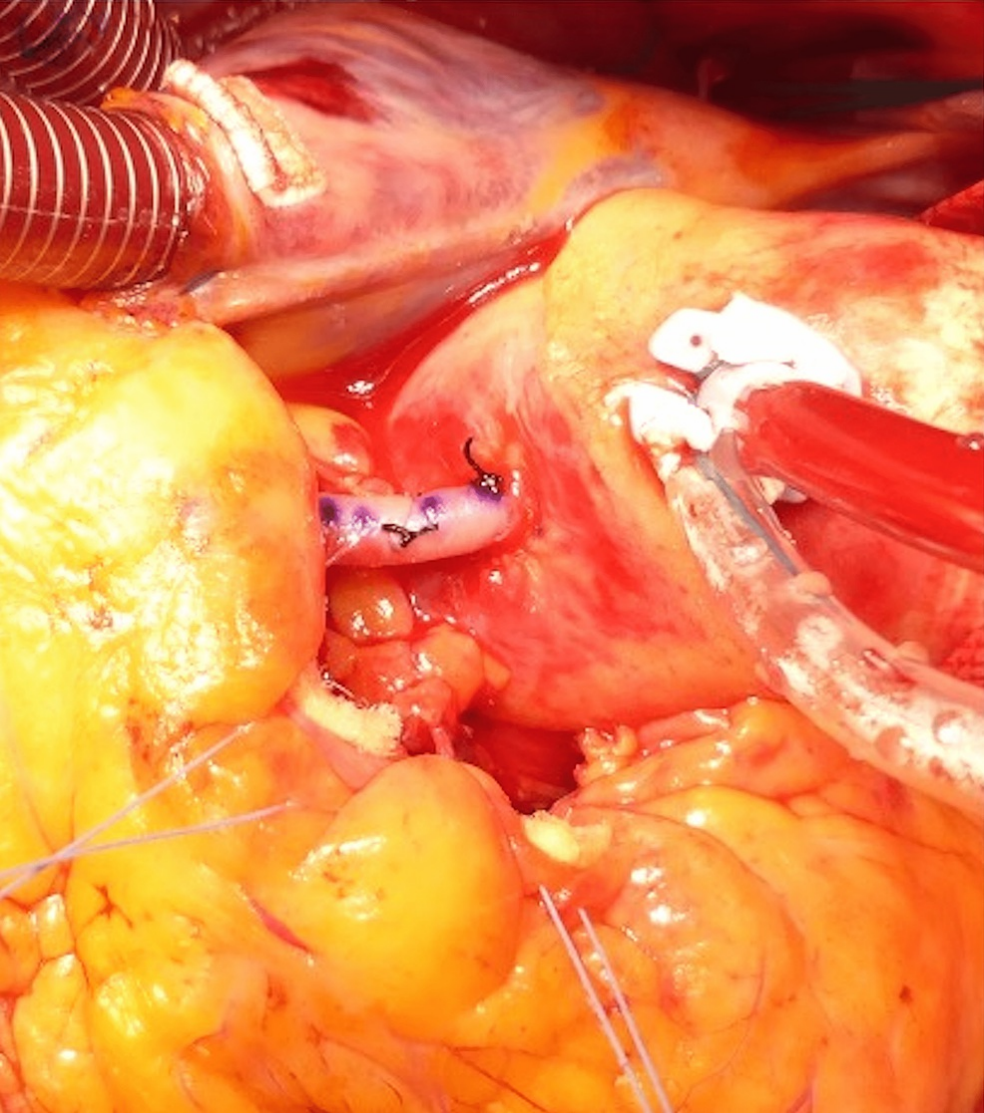
Intraoperative photograph A marking is placed on the sinotubular junction of the right sinus of Valsalva using transesophageal echocardiography, the site is punched out, and a 7-0 polypropylene suture is used for continuous anastomosis.

Exercise technetium-loaded myocardial scintigraphy revealed a decreased accumulation in the inferior wall. The patient was diagnosed with AAORCA from the left sinus of Valsalva, with right coronary ostial stenosis. Given the unpredictable natural history of the condition, the patient’s age, and evidence of stress-induced myocardial ischemia, the patient underwent cardiac surgery. A median sternotomy was performed. Cardiopulmonary bypass via the ascending aorta and the superior and inferior vena cava was established. The ascending aorta was clamped and myocardial protection was obtained with an antegrade cold blood cardioplegic solution administered intermittently. The RCA was dissected at its origin, and the central resection end was closed with a 5-0 polypropylene suture. A 5-mm hole was punched in the right sinus of Valsalva to set the anastomotic opening. The location of the neo-ostium was determined using TEE, ensuring no interference with the commissure or cusps. The anastomosis was checked to ensure that it did not interfere with the commissure or cusp. After adequate mobilization, the RCA was directly reimplanted in a standard end-to-side anastomosis using 7-0 polypropylene sutures (Figures 4, 5).

**Figure 6:**
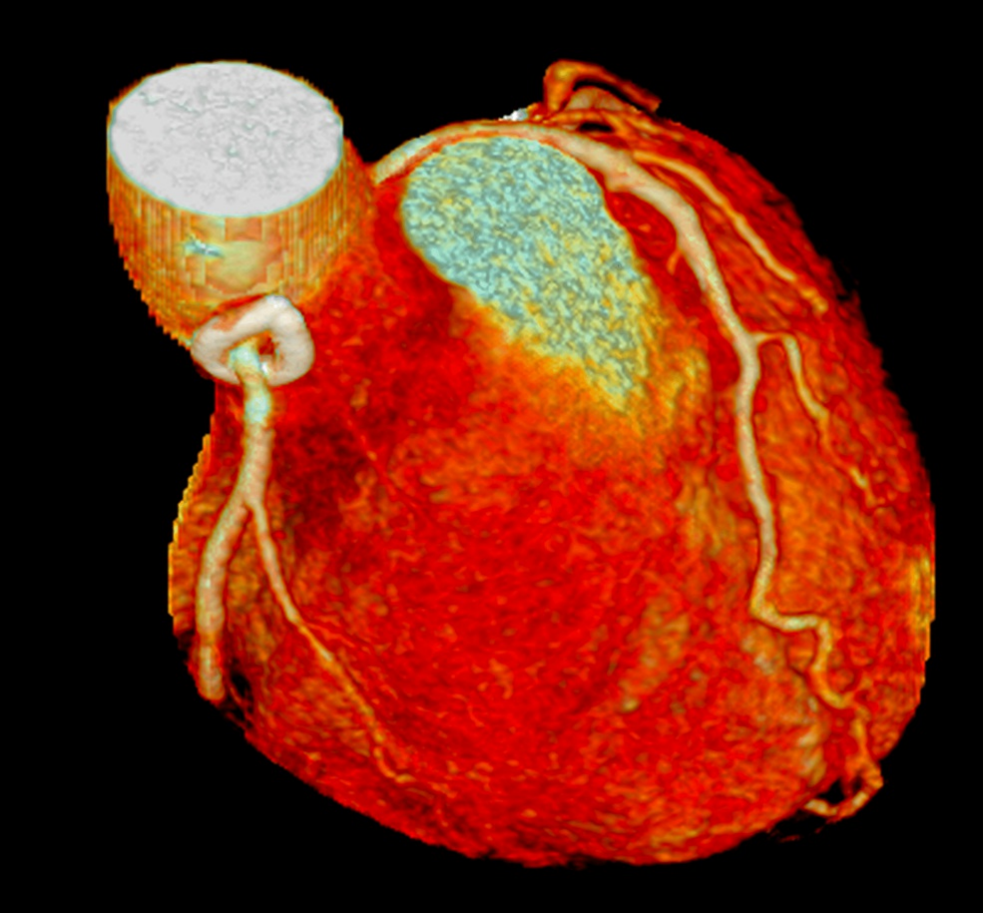
Postoperative coronary CT Postoperative coronary CT confirms the absence of kinking, stenosis, and stretching of the right coronary artery.

The aortic cross-clamp and cardiopulmonary bypass times were 63 and 111 minutes, respectively. The anastomosis was confirmed to be normal coronary artery blood flow in diastole using a flow meter. Postoperative CT angiography confirmed the patency of the RCA from the new anastomotic orifice (Figure 6).

Postoperative transthoracic echocardiogram showed normal cardiac function. Furthermore, normal aortic valvular motion was confirmed without aortic regurgitation. The patient had an uneventful postoperative course, with complete symptom relief. The patient remained asymptomatic for one year.

## Discussion

Although AAOCA is associated with angina, syncope, palpitations, and sudden cardiac death, most patients might remain asymptomatic throughout life. Diagnosing AAOCA in asymptomatic patients is extremely difficult. Moreover, AAOCA is most often detected incidentally on coronary CT or angiography performed at the time of symptom onset. In older patients, the condition can remain unnoticed until presenting with myocardial infarction due to atherosclerosis. Therefore, it is important to determine the appropriate indication for surgery in patients with incidentally diagnosed AAOCA and perform prompt surgical intervention. The American Association of Thoracic Surgeons expert consensus guidelines published in 2017 recommend surgery for AAORCA patients with chest symptoms, syncope secondary to ventricular arrhythmias, and a family history of sudden cardiac death (class I, level B) [5]. Surgery is recommended by the European Society of Cardiology for patients with AAOCA with typical angina symptoms who present with evidence of stress-induced myocardial ischemia (class I, level C) [6]. Ischemic myocardial volume, which considers the degree and extent of ischemia, correlates better with prognosis than the severity of coronary artery lesions and has a strong correlation with events such as nonfatal myocardial infarction and cardiac death [7]. Since stress myocardial scintigraphy demonstrated induced ischemia consistent with the dominant RCA region, the patient underwent surgery. The mechanisms underlying myocardial ischemia and sudden cardiac death in patients with AAORCA are still poorly understood. Cheitlin et al. proposed that during exercise, increased blood flow causes the aorta to dilate, compressing the coronary arteries that pass between it and the pulmonary artery, impeding blood flow and possibly causing myocardial ischemia [8]. Furthermore, it has been suggested that a sharp opening of the coronary artery origin may lead to myocardial ischemia because the coronary arteries become slit and bend easily [9]. Surgical procedures for AAOCA include unroofing, ostioplasty, and reimplantation to avoid the risk of stenosis of the abnormal coronary arteries (class I, level B). Reimplantation can accommodate different forms of AAOCA because they are implanted in anatomically normal locations [7]. AAORCA tends to run longer in the central direction than normal, and the anastomotic orifice to be set is usually to preserve the proximal branches of the RCA. The anastomotic orifice should be reimplanted higher than the normal opening of the right sinus of Valsalva [10]. Careful attention must be paid to the setting of the anastomotic orifice, as a low anastomotic position may result in central stenosis. The most important aspect of this procedure is to estimate the exact length of the RCA.

## Conclusions

AAOCA is a rare congenital abnormality that could lead to sudden cardiac death. Appropriate imaging studies and surgery should be performed in symptomatic patients with AAORCA who have inter-arterial paths and ostial stenosis. We report a case of anomalous RCA that traveled between the ascending aorta and pulmonary artery with right coronary ostial stenosis. Therefore, direct reimplantation in AAORCA is a simple surgical technique that normalizes anatomy and physiology. The most important aspect of this procedure is to estimate the exact length of the RCA.
